# Post-menopausal Women Exhibit Greater Interleukin-6 Responses to Mental Stress Than Older Men

**DOI:** 10.1007/s12160-016-9783-y

**Published:** 2016-03-04

**Authors:** Romano Endrighi, Mark Hamer, Andrew Steptoe

**Affiliations:** Department of Medical and Clinical Psychology, Uniformed Services University of the Health Sciences, Bethesda, MD USA; National Centre Sport and Exercise Medicine, Loughborough University, Loughborough, UK; Department of Epidemiology and Public Health, University College London, London, UK; Division of Behavioral Science Research, Department of Health Policy and Health Services Research, Boston University, Henry M. Goldman School of Dental Medicine, 560 Harrison Ave, 3rd floor, Boston, MA 02118 USA

**Keywords:** Sex, Mental stress, Pro-inflammatory reactivity, Interleukin-6

## Abstract

**Background:**

Acute stress triggers innate immune responses and elevation in circulating cytokines including interleukin-6 (IL-6). The effect of sex on IL-6 responses remains unclear due to important limitations of previous studies.

**Purpose:**

The purpose of this study was to examine sex differences in IL-6 responses to mental stress in a healthy, older (post-menopausal) sample accounting for several moderating factors.

**Methods:**

Five hundred six participants (62.9 ± 5.60 years, 55 % male) underwent 10 min of mental stress consisting of mirror tracing and Stroop task. Blood was sampled at baseline, after stress, and 45 and 75 min post-stress, and assayed using a high sensitivity kit. IL-6 reactivity was computed as the mean difference between baseline and 45 min and between baseline and 75 min post-stress. Main effects and interactions were examined using ANCOVA models.

**Results:**

There was a main effect of time for the IL-6 response (*F*_3,1512_ = 201.57, *p* = <.0001) and a sex by time interaction (*F*_3,1512_ = 17.07, *p* = <.001). In multivariate adjusted analyses, IL-6 reactivity was significantly greater in females at 45 min (*M* = 0.37 ± 0.04 vs. 0.20 ± 0.03 pg/mL, *p* = .01) and at 75 min (*M* = 0.57 ± 0.05 vs. 0.31 ± 0.05 pg/mL, *p* = .004) post-stress compared to males. Results were independent of age, adiposity, socioeconomic position, depression, smoking and alcohol consumption, physical activity, statin use, testing time, task appraisals, hormone replacement, and baseline IL-6. Other significant predictors of IL-6 reactivity were lower household wealth, afternoon testing, and baseline IL-6.

**Conclusions:**

Healthy, post-menopausal females exhibit substantially greater IL-6 responses to acute stress. Inflammatory responses if sustained over time may have clinical implications for the development and maintenance of inflammatory-related conditions prevalent in older women.

Psychosocial stress is a risk factor for cardiovascular disease (CVD) and depressive disorders [1, 2]. Mechanisms mediating the adverse effect of stress on CVD risk include exaggerated or dysregulated immune activation in response to psychosocial stress [1, 3]. Acute mental stress elicited using behavioral or cognitive tasks is known to up-regulate pro-inflammatory gene expression in peripheral mononuclear blood cells resulting in the synthesis and release of inflammatory cytokines [4, 5]. Evidence shows that the binding activity of the nuclear factor-kappaB (NF-kB), a key mediator of inducible transcription in the immune system and inflammatory genes, is enhanced by exposure to acute stress in humans. This is likely mediated through adrenergic receptor stimulation [4, 6, 7] and appears to be inversely related to glucocorticoid responses [7, 8].

In humans, elevation in inflammatory cytokines including interleukin-6 (IL-6) and interleukin-1 beta (IL-1β) can be detected in peripheral blood as early as 30 min following stress and may continue to rise up to 2 h and more after stress exposure [9]. Interestingly, there appear to be robust individual differences in the magnitude and pattern of inflammatory stress responses [8, 9]. In addition, inflammatory responses do not seem to habituate to repeated exposure to acute stress; in other words, inflammatory responses sensitize [10, 11]. This suggests that over time, continued exposure to stress-induced inflammatory responses to psychosocial stress may up-regulate low-grade, peripheral inflammation. Up-regulation of inflammatory activity may ultimately lead to greater risk of developing inflammatory-related diseases or the exacerbation of existing ones. However, apart from two studies showing positive associations between stress-induced inflammatory responses at baseline and inflammation-related CV risk factors 3 years later [3, 12], prospective evidence supporting the hypotheses of inflammatory responses linked to inflammation-related disease risk is scarce.

Sex differences in pro-inflammatory responses to acute mental stress have been reported in some studies, but the evidence is inconclusive. Greater IL-6 and interleukin-1 receptor antagonist (IL-1Ra) responses in females 45 min post-stress compared to males [13] have been previously observed. However, null and contradictory findings have also been reported. Stress-induced IL-6 responses to the paced auditory serial addition task were significantly greater in males 30 min post-stress in a small study of individuals aged 21 years on average [14]. Yet, no differences were observed at 60 min post-stress exposure [14].

Pro-inflammatory cytokine production has also been examined by evaluating changes in vitro following stimulation of white blood cells using immunogens such as lipopolysaccharide (LPS) [9]. Although in vitro responses and changes in non-stimulated levels likely involve different mechanisms, examining sex differences in stimulated inflammatory responses may also be relevant. A study of inflammatory responses to LPS in 294 young individuals [15] reported no sex differences in stimulated IL-6 reactivity. However, males had higher C-reactive protein (CRP) responses compared to females. Yet, another study in an older sample found no sex differences in stimulated IL-6 responses to a 5-min speech task 30 min post-stress [16]. However, in a subgroup analysis the authors observed that post-menopausal females had greater IL-6 responses compared to pre-menopausal females. This finding indicates that menopausal status may account for some of the inconsistencies observed among previous studies. Reasons for these inconsistencies may also be attributable to differences in the stress stimuli used (socially evaluative versus cognitive or in vitro stimulation), small sample sizes that do not allow sex-specific analyses, inadequate or limited post-stress blood sampling, and failure to include important covariates.

Sex differences in inflammatory stress responsivity may be expected because, in general, females exhibit greater sympathoadrenal responsiveness compared to males [17]. In addition, given that sex hormones are implicated in the glucocorticoid response to acute stress, and that females generally produce greater stress hormones when in the luteal phase of the menstrual cycle [18], changes in hormonal output associated with aging suggest that sex difference in inflammatory stress responses may more consistently emerge in older participants. Heightened pro-inflammatory stress responses may therefore be a relevant sex-specific risk marker in middle- and older-aged individuals, but most previous studies have mainly included young subjects.

Since several inflammatory-related conditions are more prevalent in older populations [19, 20], identifying sex differences in pro-inflammatory stress responses in the post-menopausal stage may be important for preventive strategies. Accordingly, the aim of this analysis was to examine sex differences in IL-6 responses to mental stress in a large sample of healthy, post-menopausal women, and men. We hypothesized that (I) mild behavioral stress would elicit substantial IL-6 increases between baseline and 75 min post-stress, and (II) females would exhibit significantly greater IL-6 responses throughout the stress protocol compared to males, and (III) this sex difference would survive adjustment for relevant covariates that are related to either sex or inflammatory stress responses.

## Methods

### Participants’ Characteristics

Healthy participants drawn from the Whitehall II epidemiological cohort [21] were invited to take part in a parent study examining the role of psychophysiologic factors in CVD risk [22]. Exclusion criteria included objective signs of clinical or subclinical CVD, cancer treatment in the past 5 years, regular use of prescribed medications (excluding statins), current hypertension, any inflammatory or autoimmune conditions, and diagnosed mental health disorders. These criteria were verified through review of clinical data from previous phases of the Whitehall II study. Participant selection was stratified by the latest grade of employment to ensure adequate inclusion of individuals of higher and lower socioeconomic position. Potentially eligible participants were identified and screened to determine final eligibility, yielding a sample of 543 individuals aged 53–76 years who underwent acute mental stress.

Complete data on outcome (IL-6) was available for 506 participants, but no significant differences were observed in age, BMI, depressive symptoms, socioeconomic position, or frequency of physical activity between participants with complete IL-6 data (*n* = 506) and those excluded (*n* = 37) (*P* value range 0.5 to 0.99).

Ethical approval was granted by the joint University College London/University College Hospital Ethics Committee on the ethics of human research, and all participants signed an informed consent form.

### Materials

Data on socio-demographics, behavioral, and health-related factors were obtained via a questionnaire that was mailed prior to the research appointment.

### Acute Mental Stress

Mental stress was elicited using two previously described standardized tasks administered under time pressure that reliably perturbs the cardiovascular and immune system [22, 23]. Briefly, in the mirror tracing task, participants were required to trace the narrow contour of a star using an electronic stylus while looking at the star’s reflection in a mirror. The apparatus emits a loud beep every time the stylus diverged from the marked edges of the star signaling an error. To sustain demand, participants were informed that average performance is completing five laps in the time allowed with a minimum number of errors.

In the modified Stroop task, participants had to select the color of a target color word printed in a discordant color (e.g., the word green printed in blue) presented in the center of a computer screen among four color choices at the bottom of the screen. Speed of presentation of the stimuli was titrated against individual performance. Participants were instructed not to talk during the stress task. Subjective stress and task engagement ratings were also obtained on a series of 7-point Likert scales anchored at 1 = not at all and 7 = very much after completing each task.

### Biochemical Assay

Peripheral blood was drawn into Vacutainer tubes containing EDTA as anticoagulant and was immediately centrifuged at 1246×*g* at room temperature for 10 min. The resulting supernatant was aliquoted in cryo vials and stored at −80 °C until assay. Samples were analyzed in duplicate using high-sensitivity enzyme-linked immunosorbent assay kits (R&D System, Oxford, UK) characterized by a minimum detectable dose range of 0.016–0.110 pg/mL and intra/inter-assay coefficients of variation of 7 and 7.2 %, respectively. IL-6 concentrations were determined with a microplate reader (Molecular Devices, UK) and SoftMax Pro 5 software using a four-parameter logistic curve-fit reduction of the raw absorbance data in accordance with the protocol.

### Covariates

Following a review of the literature, several factors known to relate to either basal or stimulated levels of IL-6 have been selected: age [13], the latest grade of employment derived from the British Civil Service classification (categorized as high, intermediate, low), and total household income (in GBP categorized as <20K, 20–40K, >40K) as an index of socioeconomic position [13, 24]. An adiposity component was computed using participants’ BMI, waist circumference, and fat mass [10, 13]. Mood symptoms were assessed with the Center for Epidemiologic Study Depression (CES-D) Scale [25], and scores were dichotomized into <16 or ≥16 (no/little risk and at risk for depression) [26]. Frequency of physical activity [27] categorized as none, ≤2/week, and ≥3/week was assessed using a validated questionnaire as previously described [28].

Time of testing (morning or afternoon) [9, 29] and post-stress ruminative thoughts (“I have been thinking about embarrassing moments during the tasks” 1 = not at all to 7 = extremely) [30] were also selected.

In addition, the following variables may potentially affect inflammatory responses and were included as covariates: smoking status (yes/no); alcohol use in the week prior to study appointment (categorized as none, below, and above recommended level); subjective task stress and engagement ratings, statin drug use (yes\no), hormone replacement therapy use (yes/no), and resting IL-6 levels.

### Procedure

To facilitate participants’ scheduling, research sessions were carried out at 09:00 a.m. or 01:00 p.m. On the appointment day, a research nurse ensured compliance with the study protocol (abstain from smoking, caffeine, alcohol, and exercise) and confirmed absence of acute infections such as colds. Participant’s height and weight were measured using a stadiometer and a Tanita digital scale, and BMI (kg/m^2^) was computed. Waist circumference was measured using a metal anthropometric tape midway between the lowest rib and iliac crest. Fat mass was estimated with a segmental body composition analyzer (Tanita BC-418) using bioelectrical impedance analysis. Participants were subsequently escorted to a light- and temperature-controlled stress laboratory where a venous cannula was inserted into the antecubital fossa of the non-dominant arm for repeated blood sampling. Following a 30-min rest, a 2-mL baseline blood sample was drawn into EDTA containing Vacutainer and the cannula was flushed with saline solution. Mental stress was then administered with the researcher present in the room; a second blood sample was drawn immediately after the two tasks. Participants entered the stress recovery period and were allowed to read nature magazines or watch nature DVDs. A third blood sample was obtained at 45 min into this recovery period, and the final sample was drawn at the end of the session (75 min after acute stress).

### Statistical Analyses

Based on an expected sex difference in IL-6 response of small magnitude (*f*^2^ = 0.03) [13] with power (1-β) = 0.90 and *α* = 0.05 and 11 covariates, a sample size of *n* = 460 would be required to detect a significant sex by time interaction. IL-6 data were moderately skewed and a square root transformation was used in analyses; values in tables and figures are presented in picograms per milliliter to facilitate comparability with other studies. The rumination and subjective stress scores were missing for two participants, and the CES-D score was missing for one participant; values were imputed using the mean substitution method.

Sample characteristics are presented by sex; continuous variables were compared with one-way ANOVA and categorical variables were compared with Chi square. Product–moment correlations between baseline IL-6 and sociodemographic, psychosocial, and behavioral variables were computed using Pearson’s *r* correlation coefficient.

A two (male–female) by four (baseline, after stress, and 45- and 75-min post-stress) mixed ANOVA was used to determine main effects and interactions. Significant interactions were examined using MANCOVA. In this model, two IL-6 stress reactivity scores were computed and treated as outcomes. The first score is the mean difference between the 45 min post-stress and the baseline sample (∆IL6-45 m); the second score is the mean difference between the 75 min post-stress and the baseline sample (∆IL6-75 m). Higher reactivity scores indicate greater pro-inflammatory responses to acute stress. All variables listed above were entered as covariates. Results are presented as means and standard deviations (M ± SD) or *n* and % as appropriate.

## Results

### Sociodemographic and Behavioral Characteristics of the Sample

Table 1 indicates that females were slightly older and had higher glycated hemoglobin levels than males and were also significantly less likely to engage in physical activity (*χ*^2^ = 23.28, OR = 0.21, *p* = <0.001). In contrast, males had higher systolic blood pressure, BMI, and waist circumference and were significantly more likely to be of higher employment grade (*χ*^*2*^ = 6.14, OR = 1.83, *p* = 0.013) and to have higher total household income (*χ*^*2*^ = 15.24, OR = 2.40, *p* = <0.001) compared to females. Among females, 39 (17 %) participants were on hormone replacement therapy. There were no significant sex differences in depressive symptoms, alcohol consumption, current smoking, time of stress testing, baseline subjective stress, and baseline IL-6 levels.

**Table 1:** Sociodemographic and clinical characteristics by sex (*n* = 506)

Variable	Male (*n* = 278)	Female (*n* = 228)	*p*
Age (years)	62.56 (5.80)	64.21 (5.35)	0.001
BMI (kg/m^2^)	26.36 (3.62)	25.29 (4.26)	0.002
Waist–hip ratio	0.94 (0.07)	0.81 (0.08)	<0.001
Systolic BP (mm/Hg)	127.45 (15.14)	119.82 (17.0)	<0.001
HbA1c (%)	5.40 (0.54)	5.55 (0.38)	0.001
Baseline IL-6 (pg/mL)	1.38 (0.85)	1.30 (0.81)	0.23
Baseline stress			0.09
Low	273 (98.2)	218 (95.6)	
High	5 (1.8)	10 (4.4)	
Smoking			0.88
Yes	15 (5.4)	13 (5.7)	
No	263 (94.6)	215 (94.3)	
CES-D binary			0.99
<16	251 (90.3)	205 (90.3)	
≥16	27 (9.7)	22 (9.7)	
Time of testing			0.36
Morning	114 (41)	84 (36.8)	
Afternoon	164 (59)	144 (63.2)	
Employment grade			0.01
High	116 (41.7)	85 (37.3)	
Intermediate	118 (42.4)	84 (36.8)	
Low	44 (15.8)	59 (25.9)	
Household income _GBP_			<0.001
<20K	39 (14)	64 (28.1)	
20–40K	123 (44.2)	85 (37.3)	
>40K	116 (41.7)	79 (34.6)	
Alcohol use^a^			0.28
None	39 (14.0)	43 (18.9)	
Below recommended level	203 (73.0)	153 (67.1)	
Above recommended level	36 (12.9)	32 (14.0)	
Physical activity			<0.001
None	18 (6.5)	41 (18.0)	
Up to 2/week	161 (57.9)	139 (61.0)	
3/week and over	99 (35.6)	48 (21.1)	

Values are means (SD) or *N* (%)

*BMI* body mass index, *HbA1c* glycated hemoglobin, *IL-6* interleukin-6, *CES-D* Center for Epidemiologic Study Depression Scale

^a^Recommended levels/day men = up to four units; women = up to three units

### Associations of Baseline IL-6 With Study Measures

Baseline IL-6 was significantly associated with older age (*r* = 0.2, *p* = <0.001), greater adiposity score (*r* = 0.31, *p* = <0.001), higher depression (*r* = 0.09, *p* = 0.03), lower socioeconomic status (SES; grade of employment *r* = 0.17, household income *r* = −0.17, *p* = <0.001), and lower physical activity participation (*r* = −0.1, *p* = 0.02), but was not associated with baseline stress ratings (*r* = −0.07, *p* = 0.14), current smoking (*r* = 0.07, *p* = 0.13), statin use (*r* = 0.02, *p* = 0.60), and time of stress testing (*r* = 0.06, *p* = 0.17).

### Self-Report Task Appraisal Ratings and Post-task Rumination

Males reported slightly greater average task involvement than females (5.72 ± 1.24 vs. 5.46 ± 1.50; *t*_(504)_ = 2.13, *p* = 0.03). In contrast, females perceived the stress tasks as more stressful (4.32 ± 1.50 vs. 3.96 ± 1.34, *t*_(504)_ = −2.83, *p* = 0.005) compared to males. There were no differences in post-stress ruminative thoughts (*t*_(504)_ = −1.26, *p* = 0.21).

### Pro-inflammatory Responses to Acute Mental Stress

**Fig. 1:**
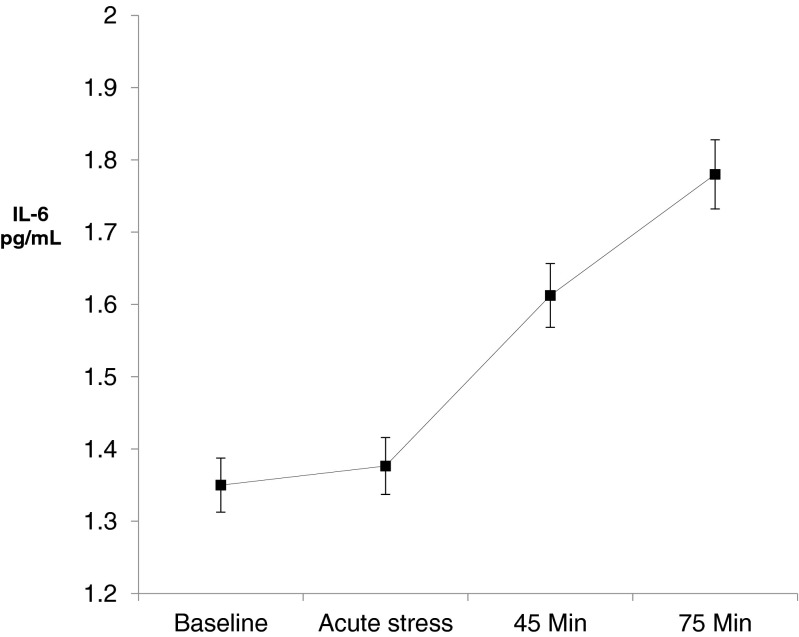
Stress-induced changes in IL-6 between baseline and the end of the stress protocol in 506 healthy, older individuals (55 % males). Values are unadjusted mean interleukin-6 expressed in picograms per milliliter at baseline (pre-stress), immediately after stress, and 45 and 75 min after stress exposure (*error bars* are standard errors of the mean). Acute stress elicited significant over time increases in serum level of IL-6 (time effect *F*_3,1512_ = 201.57, *p* = <0.001)

The IL-6 response to stress showed a significant linear increase from baseline (1.35 ± 0.83 pg/mL) to 75 min (1.78 ± 1.12 pg/mL) after the stress protocol (time effect *F*_3,1512_ = 201.57, *p* = <0.001; Fig. 1). There was also a significant sex by time interaction (*F*_3,1512_ = 17.07, *p* = <0.001) suggesting sex differences in the magnitude of this response (Table 2).

**Table 2:** Unadjusted means (SD) of pro-inflammatory IL-6 stress responses in males and females

Variable	Male (*n* = 278)	Female (*n* = 228)
Baseline	1.38 (0.85)	1.30 (0.81)
Immediately after stress	1.39 (0.86)	1.35 (0.89)
45 min after stress	1.57 (0.93)	1.69 (1.09)
75 min after stress	1.68 (1.01)	1.89 (1.22)

Values are untransformed means (standard deviations) interleukin-6 values expressed in picograms per milliliter

### Sex Differences in IL-6 Reactivity

The IL-6 reactivity scores (∆IL6-45 m and ∆IL6-75 m) were moderately well correlated with each other (*r* = 0.62, *p* = <0.001). A MANCOVA test revealed that IL-6 scores significantly differed by sex (*F*_2,488_ = 1.13, *p* = <0.001). At 45 min post-stress, females exhibited greater reactivity compared to males (∆IL6-45 m *M* = 0.144 ± 0.012 vs. 0.08 ± 0.01, *p* = <0.001). This pattern was also sustained at 75 min after stress exposure (∆IL6-75 m *M* = 0.218 ± 0.02 vs. 0.12 ± 0.01, *p* = <0.001). The fully adjusted sex difference in pro-inflammatory stress reactivity is depicted in Fig. 1.

Other statistically significant factors in the multivariate model were time of testing (*F*_2,488_ = 11.25, *p* = <0.001), indicating that participants tested in the early afternoon showed greater reactivity scores compared to participants tested in the morning, and household income (*F*_2,488_ = 2.92, *p* = 0.05), indicating that participants in the lower household income category exhibited higher reactivity scores compared to participants in the higher income categories. Baseline IL-6 was a marginally significant factor (*F*_2,488_ = 2.70, *p* = 0.06) suggesting greater reactivity in individuals with lower baseline IL-6 levels compared to those with higher levels at 75 min (*p* = 0.02) but not at 45 min post-stress (*p* = 0.16).

## Discussion

The aim of this study was to investigate sex differences in pro-inflammatory responses to acute mental stress in a large sample of healthy, older individuals and to examine whether several putative covariates moderated this effect. Consistent with most previous reports, baseline IL-6 levels were associated with older age, greater adiposity levels, and higher depressive symptoms score and inversely with socioeconomic position and frequency of physical activity [9, 10, 13]. We observed an increase (31 %) in circulating levels of IL-6 in response to mild acute stress in the whole sample, with values not returning toward baseline even at 75 min post-stress (Fig. 1).

Our main hypothesis was supported. Female participants exhibited significantly greater responses at 45 and 75 min post-stress, and this effect was independent of age, adiposity, socioeconomic position, depressive symptoms, physical activity and alcohol consumption, smoking status, lipid lowering drug use, time of testing, subjective stress and task appraisal, hormone replacement therapy use, and resting IL-6 levels. The findings reported here are consistent with a previous report that showed greater inflammatory stress responses in women 45 min post-stress [13]. Yet, the inflammatory responses observed in this study were of greater magnitude and were also sustained 75 min after stress exposure. This finding also adds support to a previous small study [16] that found sex differences in LPS-stimulated IL-6 production at 30 min post-stress between post-menopausal women compared to pre-menopausal women, and men. However, in that study, mental stress-related changes in unstimulated IL-6 levels were not observed. Furthermore, findings were not adjusted for potential confounders other than baseline IL-6.

Our results are not consistent with a previous small study [14] showing higher mental stress-induced IL-6 responses in men 30 min post-stress which was not sustained at 60 min. One explanation for this discordance is that the more robust inflammatory response and extended blood sampling implemented in our protocol made it possible to measure reliable sex effects. Of the several putative covariates examined in this study, time of testing, total household income, and, partially, baseline IL-6 levels were significant predictors in the multivariate model.

Few studies have been adequately powered to examine a wide range of factors that may theoretically be related to inflammatory reactivity. A previous study showed no associations between IL-6 level 45 min post-stress and grade of employment as an index of socioeconomic position [13]. Likewise, we observed no associations between grade of employment and inflammatory responses. However, a significant association between total household income and IL-6 responses was observed here, so that compared to the highest income group, individuals in the lowest income category had higher reactivity scores. Given that socioeconomic inequalities in CVD tend to persist into older age [31], greater pro-inflammatory responses to stress in lower SES individuals may be an important mechanism through which lower SES-related stress contributes to heightened risk of CVD.

**Fig. 2:**
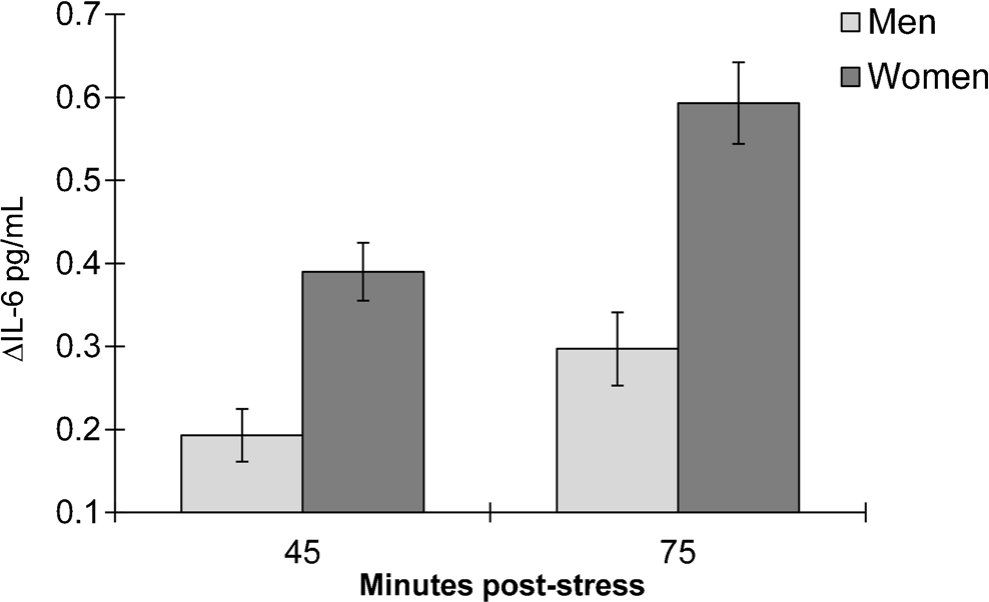
The stress-induced pro-inflammatory IL-6 response in males and females. ∆IL-6 = mean difference in blood interleukin-6 between the 45 min post-stress and baseline samples, and between the 75 min post-stress and baseline samples. Values (pg/mL) are fully adjusted for age, adiposity score, socioeconomic position, depressive symptoms, physical activity, alcohol in the past week, smoking, rumination, task appraisals, subjective stress statin drug use, hormone replacement, a.m./p.m. testing, and baseline IL-6

We predicted that the effect size of the sex difference in pro-inflammatory responses might be of small magnitude. However, even after adjusting for several covariates including baseline IL-6, the effect we observed was of medium magnitude (*f*^2^ = 0.15). Female participants produced nearly double the amount of pro-inflammatory IL-6 in response to acute stress compared to males (Fig. 2). This finding may therefore have important clinical implications. Several inflammatory-related conditions including rheumatoid arthritis and asthma [32, 33] are more prevalent in women, and stress has been identified as a trigger of symptom exacerbation. For example, emotional stress seems to exacerbate asthma symptoms through up-regulating HPA axis feedback loop leading to greater cytokine release [34, 35]. Pro-inflammatory cytokine release also plays a prominent role in negative mood symptoms and depressive disorders [36], and these conditions seem to be more common in post-menopausal women. Furthermore, prospective evidence demonstrates an association between IL-6 reactivity to stress and the development of subclinical hypertension [3]. Given that age-related hormonal changes predispose women to CVD risk, exaggerated inflammatory responses may be a mechanism underlying the association between sex and CVD risk.

Therefore, these data highlight the importance of identifying sex differences in inflammatory stress responses. Targeting stress-related responses through behavioral or pharmacological intervention aimed at reducing the magnitude of inflammatory reactivity might prove beneficial in vulnerable population such as post-menopausal women. However, it is important to note that several other factors other than inflammatory activity may contribute to the development of inflammatory-related conditions which were not addressed in this study. In addition, as noted in the “Introduction” section, there is a paucity of studies that have examined the link between inflammatory responses and disease risk prospectively. Therefore, some caution should also be used in interpreting the results showed herein given that, at present, the notion of up-regulated pro-inflammatory stress responses directly contributing to inflammatory-related disease development is not clearly supported by empirical evidence.

Several mechanisms may underlay the association between sex and inflammatory stress responses. Sex-dependent variations in estradiol hormonal levels between pre- and post-menopausal women, and men, may be responsible for the greater release of blood IL-6 in women observed in this study. Specifically, the sex effect observed might be driven by a specific inhibition of pro-inflammatory cytokine gene expression that is mediated through NF-kB binding of the hormone estradiol in post-menopausal women [37]. However, other mechanisms may also be relevant. The adipose tissue is an important source of stress-induced IL-6 release. Therefore, sex differences in the sensitivity of adipose cells to sympathetic stimulation between the sexes [38, 39] may account for the sex difference in IL-6 responses observed herein. Unfortunately, identifying the specific source of stress-induced IL-6 release would require the use of more invasive techniques that were not available in this study.

### Strengths and Limitations

Some limitations have to be highlighted. Several pro-inflammatory markers are activated by acute stress, but in this study the focus was on IL-6. It may be important to examine whether other stress-induced biomarkers also differ by sex or whether the sex effect is specific to IL-6. Our sample did not include pre-menopausal women, and it would have been informative to compare pre- and post-menopausal women, and men. To the authors’ knowledge, this is the first psychophysiologic stress study adequately powered to examine a wide range of sociodemographic, behavioral, and psychosocial factors that may confound the association of inflammatory responses with sex and to have an adequate blood sampling protocol that allowed examination of changes from baseline to up to 1 h and 15 min after stress.

We have demonstrated that female participants exhibit greater stress-induced IL-6 release, sustained to 75 min after stress exposure compared to males independent of a wide range of covariates. Sex-specific interventions to reduce the magnitude of inflammatory responses may prove beneficial in targeting stress-related symptom exacerbation in those conditions where inflammatory activity is prominent.
